# Association between timing of psychological intervention and reconstruction of worldview, life perspective, and values in adolescents with depression

**DOI:** 10.3389/fpsyt.2026.1786690

**Published:** 2026-10-05

**Authors:** Lin Chen

**Affiliations:** 1 School of Marxism, Lanzhou University, Lanzhou, Gansu, China; 2 School of Marxism, Hunan College of Humanities, Science and Technology, Loudi, Hunan, China

**Keywords:** adolescent depression, early intervention, life perspective, logistic regression, psychotherapy timing, values reconstruction, worldview reconstruction

## Abstract

**Background:**

Timely access to structured psycho-educational intervention is crucial for cognitive-motivational recovery in adolescents with depression, a population in a critical developmental stage of consolidating core belief systems. Drawing upon the theory of educational timing, the initiation of these interventions may represent a critical window for reshaping worldview, life perspective, and values. Therefore, this study aimed to examine the association between intervention timing and the reconstruction of these existential constructs, thereby providing an empirical basis for optimizing psycho-educational strategies in adolescent depression care.

**Methods:**

This retrospective cohort study included 244 adolescents with depressive disorder treated between January 2022 and December 2024. To operationalize the concept of “optimal educational timing,” the cohort was stratified based on intervention initiation: early intervention, defined as initiation of structured psycho-educational intervention within 14 days of the index clinical encounter, and delayed intervention, defined as initiation after 14 days. Outcomes were assessed using the Worldview, Life Perspective, and Values Reconstruction Questionnaire (WLVQ-22). Linear and logistic regression models were used to evaluate changes in WLVQ-22 scores and responder outcomes.

**Results:**

The cohort included 126 adolescents in the early-intervention group and 118 in the delayed-intervention group. Baseline WLVQ-22 scores were comparable between groups. After adjustment, early intervention initiation was associated with greater changes in worldview (β = 4.43, 95% confidence interval [CI]: 2.89 to 5.98; *p* < 0.001), life perspective (β = 3.93, 95% CI: 2.45 to 5.41; *p* < 0.001), and values total score (β = 2.07, 95% CI: 1.27 to 2.87; *p* < 0.001). Early intervention was also associated with higher odds of response for worldview (odds ratio [OR] = 2.96, 95% CI: 1.71 to 5.11), life perspective (OR = 2.51, 95% CI: 1.46 to 4.33), and values reconstruction (OR = 2.90, 95% CI: 1.37 to 6.13).

**Conclusions:**

Earlier initiation of structured psychological intervention was associated with more favorable short-term WLVQ-22-assessed reconstruction indicators in adolescents with depression. These findings highlight the need for prospective studies to further evaluate the role of intervention timing in adolescent depression care.

## 1 Introduction

Adolescent depression is a prevalent and clinically consequential condition that emerges during a developmental period characterized by rapid neurobiological maturation and the consolidation of identity, future orientation, and value systems (1). Recent population-based evidence indicates increasing rates of clinically diagnosed depressive disorders among youth, underscoring an escalating service need and a persistent treatment gap (2, 3). Consistently, global public health reporting highlights that depression is among the leading causes of illness and disability in adolescence and that unmet mental health needs may confer long-term functional sequelae extending into adulthood (4, 5). Contemporary quantitative syntheses also suggest a substantial global burden of depressive symptoms and depression in children and adolescents, supporting the need for developmentally informed clinical strategies that address not only symptom reduction but also psychosocial and cognitive–motivational reconstruction (6, 7). Beyond core affective symptoms, adolescent depression is commonly accompanied by distortions in meaning-making and self-referential cognition, including pessimistic expectations, diminished goal-directedness, reduced perceived agency, and impaired future-oriented thinking (8). These features may undermine the development of a coherent worldview, adaptive life perspective, and stable values during a period when these higher-order psychological systems are still being formed (9). Future-related thinking has increasingly been recognized as a transdiagnostic process relevant to depressive psychopathology in young people, with systematic review-level evidence supporting its clinical salience (10, 11). Complementary work on meaning in life among adolescents further suggests that meaning-related profiles are associated with psychological vulnerability and resilience, providing a conceptual bridge between depressive symptoms and higher-order cognitive–motivational reconstruction targets (12, 13).

Evidence-based psychological treatments for adolescent depression include structured approaches that target maladaptive beliefs, interpersonal functioning, behavioral activation, value-consistent action, and psychological flexibility (14). A growing literature supports acceptance- and values-oriented modalities, including meta-analytic evidence for acceptance and commitment therapy in adolescent depression (15). In addition, scalable delivery models, including internet-based and digitally delivered psychological interventions, have demonstrated measurable benefits for depressive symptoms in young people and may help reduce access-related delays in treatment initiation (16, 17). Nevertheless, qualitative evidence indicates that delays in seeking and receiving care among adolescents with depression remain common and are shaped by stigma, limited symptom recognition, family-level barriers, and service accessibility constraints (18). The timing of psychological intervention may be clinically relevant because earlier initiation could provide adolescents with structured support before maladaptive cognitive–emotional patterns, avoidance behaviors, and hopeless future expectations become more consolidated (19). From a developmental perspective, timely psychological engagement may be particularly important for preserving agency, goal-directedness, interpersonal connection, and value-consistent behavior during adolescence (20).

However, empirical evidence remains limited regarding whether earlier initiation of psychological intervention is associated with better reconstruction of worldview, life perspective, and values among adolescents with depression (21). In routine clinical practice, the first two weeks after the index clinical encounter often represent a practical early-care window for diagnostic clarification, initial risk assessment, family communication, treatment planning, and psychotherapy scheduling (22). To examine the robustness of this operational definition, alternative timing thresholds and continuous time-to-intervention analyses were also planned. This study aimed to examine the association between the timing of structured psychological intervention and WLVQ-22-assessed exploratory indicators of worldview, life perspective, and values reconstruction in adolescents with depression.

## 2 Methods

### 2.1 Study design

This retrospective cohort study consecutively included adolescents diagnosed with depressive disorder who received evaluation and treatment at our institution between January 2022 and December 2024. The index date was defined as the first outpatient or inpatient encounter for the current depressive episode during the study period. The exposure of interest was the timing of initiation of structured psychological intervention following the index date. Participants were classified into an early-intervention group (psychological intervention initiated within 14 days of the index date) and a delayed-intervention group (initiation >14 days). The 14-day threshold was selected *a priori* as a pragmatic operational definition of early initiation, reflecting the usual clinical interval required for diagnostic confirmation, initial treatment planning, family communication, and psychotherapy scheduling at our institution. This cutoff was not intended to represent a validated critical window for cognitive–motivational reconstruction. Psychological intervention referred to a standardized, therapist-delivered program documented in the medical record, such as cognitive behavioral therapy–based or other manualized psychotherapy, delivered in addition to usual clinical management as clinically indicated. Outcomes related to the reconstruction of worldview, life perspective, and values were extracted from structured clinical assessments performed at baseline, before intervention initiation, and at follow-up within a prespecified clinical window after intervention initiation. Because the WLVQ-22 has not been externally validated in independent adolescent depression cohorts, WLVQ-22-based outcomes were interpreted as exploratory reconstruction indicators rather than fully validated clinical endpoints. Informed consent was obtained from all participants or their legal guardians. The study was reviewed and approved by the hospital’s ethics committee and conducted in accordance with relevant guidelines and the Declaration of Helsinki. All data were anonymized prior to analysis to ensure participant confidentiality.

### 2.2 Eligibility criteria

Inclusion Criteria: Participants were eligible if they met all of the following criteria: (1) age 12–18 years at the index date; (2) a diagnosis of depressive disorder established by a qualified psychiatrist/psychologist based on routinely applied diagnostic criteria (e.g., DSM-5/ICD criteria documented in the medical record); (3) receipt of care at the study institution between January 2022 and December 2024 with a clearly defined index visit for the current episode; (4) initiation of a structured psychological intervention with a clearly recorded start date; (5) availability of baseline WLVQ-22 assessment data evaluating worldview, life perspective, and values, obtained prior to psychological intervention; and (6) availability of at least one post-intervention follow-up WLVQ-22 assessment within a prespecified clinical window sufficient for outcome evaluation.

Exclusion Criteria: Participants were excluded if any of the following applied: (1) comorbid bipolar disorder or psychotic disorder; (2) autism spectrum disorder with substantial functional impairment, or moderate-to-severe intellectual disability precluding reliable completion of assessments; (3) active substance use disorder during the observation period; (4) severe acute suicidal risk requiring emergency/crisis-only management such that structured psychological intervention initiation and follow-up assessment could not be defined; (5) major neurological disease, clinically significant cognitive impairment, or unstable medical illness affecting participation or assessment validity; (6) concurrent enrollment in interventional studies targeting psychological outcomes during the same episode; or (7) missing essential data, including unclear intervention timing, absent baseline WLVQ-22 measures, or unavailable outcome assessments required for primary analyses.

### 2.3 Outcome measures for worldview, life perspective, and values reconstruction

The WLVQ-22 was used as a structured clinical assessment tool to capture worldview, life perspective, and values-related reconstruction. Because the WLVQ-22 has not been externally validated in independent adolescent depression cohorts, WLVQ-22-based outcomes were interpreted as exploratory reconstruction indicators rather than externally validated clinical endpoints.

Worldview reconstruction: Worldview was measured at the index visit (baseline) and at the prespecified follow-up using the Worldview Reconstruction subscale of the Worldview, Life Perspective, and Values Reconstruction Questionnaire (WLVQ-22). This subscale comprises 5 self-report items rated over the past 2 weeks on a 5-point Likert scale (0–4); reverse-keyed items were recoded prior to scoring. Item content and coding rules are provided in **Supplementary Table 1**. The worldview total score was calculated as the mean of the 5 item scores and linearly transformed to a 0–100 metric using (mean item score/4) × 100, with higher scores indicating more adaptive worldview reconstruction. The primary worldview endpoint was the absolute change from baseline to follow-up (Δworldview = worldview_follow-up − worldview_baseline). Secondary worldview metrics included percent change (Δ%worldview = [Δworldview/worldview_baseline] × 100) and responder status, defined *a priori* as Δworldview ≥10 points.

Life perspective reconstruction: Life perspective was assessed at baseline and follow-up using the Life Perspective Reconstruction subscale of the WLVQ-22, consisting of 5 items with a 2-week recall period and uniform 0–4 response options; reverse-keyed items were recoded prior to score computation. The full item list is presented in **Supplementary Table 2**. The life perspective total score was computed as the mean of the 5 items and transformed to a 0–100 scale using (mean/4) × 100, where higher values indicate greater reconstruction of future orientation and life planning. The primary life perspective endpoint was the absolute change from baseline to follow-up (Δlife perspective = life perspective_follow-up − life perspective_baseline). Additional metrics included percent change (Δ%life perspective = [Δlife perspective/life perspective_baseline] × 100) and responder status, defined *a priori* as Δlife perspective ≥10 points.

Values reconstruction: Values reconstruction was evaluated at baseline and follow-up using the Values Reconstruction section of the WLVQ-22, a multidimensional module comprising 12 items across three predefined domains: responsibility, social connectedness, and self-efficacy (4 items per domain; 2-week recall; 0–4 response options). Reverse-keyed items were recoded prior to scoring, and item-level content and domain mapping are detailed in **Supplementary Table 3**. Each domain score was calculated as the mean of its four items and transformed to a 0–100 scale as (mean/4) × 100. A composite values score (values total) was calculated as the arithmetic mean of the three domain scores. The primary values endpoint was the absolute change in the composite score (Δvalues total = values total_follow-up − values total_baseline). Secondary values metrics included domain-specific absolute changes (Δresponsibility, Δsocial connectedness, and Δself-efficacy). Responder status was defined *a priori* as Δvalues total ≥10 points. An exploratory attainment indicator was also recorded as achieving a follow-up values total score ≥60.

### 2.4 Data collection

Demographic, clinical, treatment, and outcome data were retrospectively extracted from the institutional electronic medical record system and psychological counseling documentation for all eligible adolescents with depression treated between January 2022 and December 2024. Outcome assessments were conducted as part of routine clinical care and were not performed specifically for the present timing-based research hypothesis. At the time of routine assessment, assessors were not informed of the early- versus delayed-intervention grouping for the current analysis, and participants were not informed that intervention timing would be analyzed as a study exposure. At the index visit, defined as the first qualifying clinical encounter within the study window, age, sex, school grade/educational stage, family structure, and a family support indicator derived from intake interviews and caregiver reports were collected. Baseline clinical variables included depression severity assessed using a standardized depression rating scale routinely documented at intake, episode duration, first-episode versus recurrent status, comorbid anxiety based on a standardized anxiety scale score and/or clinician diagnosis, and sleep problems based on standardized sleep assessment and/or documented insomnia symptoms.

Treatment-related variables included concurrent pharmacotherapy, medication class recorded in prescribing notes, and follow-up duration, calculated from the index visit to the scheduled post-intervention reassessment. Exposure ascertainment focused on intervention timing, operationalized as time-to-intervention in days from the index visit to the first session of protocol-consistent psychological intervention. The primary grouping used the prespecified 14-day cutoff to define early versus delayed initiation.

WLVQ-22-based outcomes were abstracted from structured patient-reported or clinician-administered assessments completed at baseline and follow-up, capturing three prespecified constructs: worldview reconstruction, life perspective reconstruction, and values reconstruction. Change outcomes were calculated as absolute change, defined as follow-up minus baseline, and percent change relative to baseline. Responder status was defined *a priori* using prespecified improvement thresholds. Data extraction was conducted using a standardized case report form, and a subset of records underwent independent verification to minimize abstraction errors. All data were de-identified before analysis. Missingness in key exposure or outcome fields resulted in exclusion from the analytic cohort according to the predefined eligibility and case-completeness criteria.

### 2.5 Statistical analysis

All analyses were performed using IBM SPSS Statistics, version 26.0. Continuous variables were summarized as mean ± standard deviation or median with interquartile range, and categorical variables as counts and percentages. Between-group comparisons were conducted using the independent-samples t test, Mann–Whitney U test, chi-square test, or Fisher’s exact test, as appropriate. Baseline covariate balance was assessed using absolute standardized mean differences (SMDs), with values <0.10 considered acceptable. Changes in WLVQ-22 scores were calculated as follow-up minus baseline. Linear regression was used for continuous change outcomes, and logistic regression was used for responder outcomes. Estimates were reported as β coefficients or odds ratios (ORs) with 95% confidence intervals (CIs). Adjusted models included age, sex, baseline PHQ-9 score, episode duration, first-episode status, comorbid anxiety, sleep problems, concurrent pharmacotherapy, family structure/family support indicator, follow-up duration, and the corresponding baseline WLVQ score. Sensitivity analyses were performed using 7-day, 14-day, and 21-day intervention-timing cutoffs, follow-up restriction to 6–10 weeks, inverse probability of treatment weighting, propensity score matching, and continuous time-to-intervention modeled per 7-day delay. Covariate balance after propensity score adjustment was evaluated using SMDs. Exploratory psychometric analyses of the WLVQ-22 included Cronbach’s α, corrected item-total correlations, correlations with baseline PHQ-9 scores, and standardized response means. All tests were two-sided, with *p* < 0.05 considered statistically significant.

## 3 Results

### 3.1 Study flow and participant selection

This retrospective cohort study screened adolescents with depressive disorder using institutional electronic medical records and routine psychological assessment data from January 1, 2022 to December 31, 2024. A total of 257 patients were initially identified. After eligibility assessment, 13 patients were excluded because of missing key data, major psychiatric comorbidities, acute high suicide risk requiring crisis-only management, cognitive/developmental impairment, or severe medical conditions compromising assessment validity. The final analytic cohort included 244 adolescents, including 118 in the delayed-intervention group and 126 in the early-intervention group.

### 3.2 Baseline characteristics

Baseline demographic, clinical, and treatment-related characteristics are summarized in **Table 1**. The mean age was 14.57 ± 1.34 years in the delayed-intervention group and 14.90 ± 1.33 years in the early-intervention group (*p* = 0.052). No statistically significant between-group differences were observed for sex, school grade, family structure, PHQ-9 score, episode duration, first-episode status, comorbid anxiety, sleep problems, concomitant pharmacotherapy, medication class, fluoxetine-equivalent dose, or follow-up duration (all P > 0.05). However, several variables showed absolute standardized mean differences greater than 0.10, including age, sex, senior grade, family structure, first-episode status, sleep problems, medication class, and fluoxetine-equivalent dose. These variables were included or considered in the adjusted and propensity score-based analyses where applicable.

**Table 1:** Baseline demographic, clinical, and treatment characteristics of the study cohort.

Variable	Delayed intervention (n=118)	Early intervention (n=126)	Test statistic	*P* value	Absolute SMD
Age, years	14.57 ± 1.34	14.90 ± 1.33	t = −1.95	0.052	0.247
Male sex, n (%)	56 (47.5)	69 (54.8)	χ² = 1.30	0.254	0.147
Senior grade, n (%)	43 (36.4)	59 (46.8)	χ² = 2.70	0.100	0.212
Family structure, n (%)			χ² = 3.59	0.166	0.240
Two-parent family	89 (75.4)	82 (65.1)			
Single-parent family	21 (17.8)	35 (27.8)			
Other guardian	8 (6.8)	9 (7.1)			
PHQ-9 score	16.20 ± 4.42	15.92 ± 4.29	t = 0.51	0.612	0.064
Episode duration, months	7.0 [5.0, 10.5]	7.0 [4.6, 9.2]	U = 7616	0.742	Not estimated
First episode, n (%)	76 (64.4)	68 (54.0)	χ² = 2.75	0.098	0.214
Comorbid anxiety, n (%)	52 (44.1)	60 (47.6)	χ² = 0.31	0.578	0.071
Sleep problems, n (%)	62 (52.5)	75 (59.5)	χ² = 1.21	0.272	0.141
Concomitant pharmacotherapy, n (%)	66 (55.9)	72 (57.1)	χ² = 0.04	0.849	0.024
Medication class among treated, n (%)			χ² = 0.68	0.411	0.141
SSRI	55 (83.3)	56 (77.8)			
SNRI	11 (16.7)	16 (22.2)			
Fluoxetine-equivalent dose, mg/day‡	20.80 ± 6.30	21.96 ± 5.81	t = −1.13	0.261	0.192
Follow-up duration, weeks	7.95 ± 1.81	8.00 ± 1.98	t = −0.23	0.822	0.026

### 3.3 Psychometric properties of the WLVQ-22

Exploratory psychometric analyses of the WLVQ-22 are presented in **Supplementary Table 4**. Internal consistency was acceptable for the worldview reconstruction subscale (Cronbach’s α = 0.84), life perspective reconstruction subscale (α = 0.86), values reconstruction total scale (α = 0.91), and WLVQ-22 total score (α = 0.93). Corrected item-total correlations ranged from 0.46 to 0.71 for worldview reconstruction, 0.49 to 0.74 for life perspective reconstruction, and 0.43 to 0.76 for values reconstruction. Baseline WLVQ-22 scores were negatively correlated with baseline PHQ-9 scores, with correlation coefficients ranging from −0.36 to −0.57 (all *p* < 0.001). Standardized response means ranged from 1.28 to 2.15 across WLVQ-22 domains, suggesting preliminary responsiveness to longitudinal change within the present cohort (**Supplementary Table 4**).

### 3.4 Worldview reconstruction outcomes

Worldview scores at baseline and follow-up are shown in **Table 2**. Baseline worldview scores did not differ significantly between the delayed- and early-intervention groups (46.9 ± 8.1 vs 48.2 ± 9.6; *p* = 0.278). At follow-up, the early-intervention group had higher worldview scores than the delayed-intervention group (61.2 ± 11.9 vs 55.7 ± 9.8; *p* < 0.001). Greater absolute and percent changes in worldview score were observed in the early-intervention group than in the delayed-intervention group (absolute change: 13.0 ± 6.3 vs 8.8 ± 5.8; *p* < 0.001; percent change: 28.1% ± 14.8% vs 19.4% ± 14.0%; *p* < 0.001). The proportion of worldview responders was higher in the early-intervention group than in the delayed-intervention group (66.7% vs 43.2%; *p* < 0.001) (**Table 2**).

**Table 2:** Worldview scores at baseline and follow-up, and change metrics by intervention timing group.

Outcome	Delayed (n=118)	Early (n=126)	Test statistic	*P* value
Worldview score at baseline	46.9 ± 8.1	48.2 ± 9.6	t = -1.09	0.278
Worldview score at follow-up	55.7 ± 9.8	61.2 ± 11.9	t = -3.92	<0.001
Absolute change (Δ)	8.8 ± 5.8	13.0 ± 6.3	t = -5.46	<0.001
Percent change, %	19.4 ± 14.0	28.1 ± 14.8	t = -4.69	<0.001
Responders (Δ ≥ 10), n (%)	51 (43.2)	84 (66.7)	χ² = 13.55	<0.001

Δ, absolute change from baseline to follow-up; SD, standard deviation.

In regression analyses, the unadjusted between-group difference in Δworldview was 4.25 points (95% CI: 2.72 to 5.79; *p* < 0.001). After adjustment, early intervention initiation remained associated with greater Δworldview (β = 4.43, 95% CI: 2.89 to 5.98; *p* < 0.001). The adjusted odds ratio for worldview response was 2.96 (95% CI: 1.71 to 5.11; *p* < 0.001) (**Table 3**).

**Table 3:** Between-group differences in worldview improvement.

Analysis	Estimate	95% CI	*P* value
Unadjusted between-group difference in Δworldview, Early − Delayed	β = 4.25	2.72 to 5.79	<0.001
Adjusted effect on Δworldview, coefficient for Early	β = 4.43	2.89 to 5.98	<0.001
Unadjusted responder risk difference, Early − Delayed	23.40%	11.3% to 35.6%	<0.001
Unadjusted responder odds, Early vs Delayed	OR = 2.63	1.56 to 4.42	<0.001
Adjusted responder odds, Early vs Delayed	OR = 2.96	1.71 to 5.11	<0.001

### 3.5 Life perspective reconstruction outcomes

Life perspective outcomes are summarized in **Table 4**. Baseline life perspective scores were similar between the delayed- and early-intervention groups (42.7 ± 7.8 vs 43.9 ± 9.3; *p* = 0.294). At follow-up, the early-intervention group had higher life perspective scores than the delayed-intervention group (55.1 ± 11.6 vs 50.2 ± 9.4; *p* < 0.001). Greater absolute and percent changes in life perspective score were observed in the early-intervention group than in the delayed-intervention group (absolute change: 11.2 ± 6.0 vs 7.5 ± 5.6; *p* < 0.001; percent change: 26.7% ± 15.8% vs 18.3% ± 14.8%; *p* < 0.001). The responder proportion was also higher in the early-intervention group (55.6% vs 36.4%; *p* = 0.003) (**Table 4**).

**Table 4:** Life perspective scores at baseline and follow-up, and change metrics by intervention timing group.

Outcome	Delayed (n=118)	Early (n=126)	Test statistic	*P* value
Life perspective score at baseline	42.7 ± 7.8	43.9 ± 9.3	t = -1.05	0.294
Life perspective score at follow-up	50.2 ± 9.4	55.1 ± 11.6	t = -3.61	<0.001
Absolute change (Δ)	7.5 ± 5.6	11.2 ± 6.0	t = -5.01	<0.001
Percent change, %	18.3 ± 14.8	26.7 ± 15.8	t = -4.29	<0.001
Responders, Δ ≥10, n (%)	43 (36.4)	70 (55.6)	χ² = 8.95	0.003

Δ, absolute change; SD, standard deviation.

### 3.6 Values reconstruction outcomes

Values reconstruction outcomes are presented in **Table 5**. Baseline scores for responsibility, social connectedness, self-efficacy, and total values score did not differ significantly between groups. At follow-up, the early-intervention group had a higher total values score than the delayed-intervention group (66.2 ± 7.1 vs 64.1 ± 7.2; *p* = 0.018). A greater increase in total values score was observed in the early-intervention group (9.3 ± 3.6 vs 6.8 ± 3.5; *p* < 0.001). Among the values domains, between-group differences in change were observed for social connectedness (11.0 ± 5.8 vs 7.0 ± 6.6; *p* < 0.001) and self-efficacy (8.9 ± 6.3 vs 6.5 ± 5.5; *p* = 0.002), but not for responsibility (7.8 ± 5.2 vs 6.9 ± 5.8; *p* = 0.172). The values responder proportion was higher in the early-intervention group than in the delayed-intervention group (44.4% vs 17.8%; *p* < 0.001). Attainment of a follow-up total values score ≥60 was 81.7% in the early-intervention group and 71.2% in the delayed-intervention group (*p* = 0.051) (**Table 5**).

**Table 5:** Values reconstruction dimension scores at baseline and follow-up, with between-group comparisons.

Outcome	Delayed (n=118)	Early (n=126)	Test statistic	*P* value
Responsibility at baseline	57.5 ± 10.0	57.7 ± 8.7	t = -0.16	0.877
Responsibility at follow-up	64.4 ± 10.3	65.5 ± 9.2	t = -0.93	0.356
Responsibility change (Δ)	6.9 ± 5.8	7.8 ± 5.2	t = -1.37	0.172
Social connectedness at baseline	54.4 ± 10.5	54.2 ± 10.7	t = 0.16	0.875
Social connectedness at follow-up	61.5 ± 11.8	65.3 ± 11.9	t = -2.50	0.013
Social connectedness change (Δ)	7.0 ± 6.6	11.0 ± 5.8	t = -5.05	<0.001
Self-efficacy at baseline	59.8 ± 8.5	58.9 ± 8.3	t = 0.82	0.414
Self-efficacy at follow-up	66.3 ± 10.3	67.8 ± 9.9	t = -1.18	0.239
Self-efficacy change (Δ)	6.5 ± 5.5	8.9 ± 6.3	t = -3.16	0.002
Total values score at baseline	57.3 ± 6.8	56.9 ± 6.1	t = 0.37	0.711
Total values score at follow-up	64.1 ± 7.2	66.2 ± 7.1	t = -2.37	0.018
Total values score change (Δ)	6.8 ± 3.5	9.3 ± 3.6	t = -5.40	<0.001
Responders (Δtotal ≥ 10), n (%)	21 (17.8)	56 (44.4)	χ² = 20.03	<0.001
Attainment (follow-up total ≥ 60), n (%)	84 (71.2)	103 (81.7)	χ² = 3.80	0.051

Δ, absolute change from baseline to follow-up; SD, standard deviation.

### 3.7 Adjusted associations between intervention timing and reconstruction outcomes

**Table 6** summarizes the unadjusted and adjusted associations between intervention timing and the reconstruction outcomes. In linear regression models, early intervention initiation was associated with greater changes in worldview (adjusted β = 4.43, 95% CI: 2.89 to 5.98; *p* < 0.001), life perspective (adjusted β = 3.93, 95% CI: 2.45 to 5.41; *p* < 0.001), and values total score (adjusted β = 2.07, 95% CI: 1.27 to 2.87; *p* < 0.001). In logistic regression models, early intervention initiation was associated with higher odds of response for worldview (adjusted OR = 2.96, 95% CI: 1.71 to 5.11; *p* < 0.001), life perspective (adjusted OR = 2.51, 95% CI: 1.46 to 4.33; *p* < 0.001), and values reconstruction (adjusted OR = 2.90, 95% CI: 1.37 to 6.13; *p* = 0.005) (**Table 6**).

**Table 6:** Unadjusted and adjusted associations between intervention timing and outcome changes.

Outcome	Model type	Unadjusted β/OR	95% CI	*P* value	Adjusted β/OR	95% CI	*P* value
Δworldview	Linear regression	β = 4.25	2.72 to 5.79	<0.001	β = 4.43	2.89 to 5.98	<0.001
Δlife perspective	Linear regression	β = 3.74	2.27 to 5.21	<0.001	β = 3.93	2.45 to 5.41	<0.001
Δvalues total	Linear regression	β = 2.46	1.57 to 3.36	<0.001	β = 2.07	1.27 to 2.87	<0.001
Worldview responder	Logistic regression	OR = 2.63	1.56 to 4.42	<0.001	OR = 2.96	1.71 to 5.11	<0.001
Life perspective responder	Logistic regression	OR = 2.18	1.30 to 3.64	0.003	OR = 2.51	1.46 to 4.33	<0.001
Values responder	Logistic regression	OR = 3.70	2.05 to 6.65	<0.001	OR = 2.90	1.37 to 6.13	0.005

### 3.8 Sensitivity analyses

Sensitivity analyses are summarized in **Table 7**. Across the 7-, 14-, and 21-day cutoff definitions, earlier intervention initiation was associated with greater changes in worldview, life perspective, and values total scores. With the 7-day cutoff, the association with worldview responder status was attenuated and did not reach statistical significance (OR = 1.96, 95% CI: 0.98 to 3.90; *p* = 0.057), whereas continuous outcome changes remained statistically significant. Using the 14-day cutoff, the adjusted estimates were β = 4.43 for Δworldview, OR = 2.96 for worldview response, β = 3.93 for Δlife perspective, and β = 2.07 for Δvalues total. Using the 21-day cutoff, the corresponding estimates remained statistically significant for all listed outcomes (**Table 7**).

**Table 7:** Sensitivity analyses of the association between intervention timing and outcome changes.

Scenario	N early/delayed	Worldview Δ: β (95% CI); P	Worldview responder: OR (95% CI); P	Life perspective Δ: β (95% CI); P	Values total Δ: β (95% CI); P
Cutoff = 7 days	47/197	3.00 (0.97 to 5.04); 0.004	1.96 (0.98 to 3.90); 0.057	2.46 (0.27 to 4.65); 0.028	1.12 (0.15 to 2.09); 0.024
Cutoff = 14 days	126/118	4.43 (2.89 to 5.98); <0.001	2.96 (1.71 to 5.11); <0.001	3.93 (2.45 to 5.41); <0.001	2.07 (1.27 to 2.87); <0.001
Cutoff = 21 days	162/82	3.49 (1.83 to 5.16); <0.001	2.36 (1.35 to 4.13); 0.003	4.10 (2.51 to 5.68); <0.001	1.26 (0.46 to 2.06); 0.002
Follow-up restricted to 6–10 weeks	81/86	5.00 (3.06 to 6.94); <0.001	3.66 (1.87 to 7.18); <0.001	3.91 (1.80 to 6.02); <0.001	1.80 (0.83 to 2.77); <0.001
IPTW, stabilized and truncated	126/118	4.23 (2.67 to 5.78); <0.001	2.84 (1.67 to 4.83); <0.001	4.10 (2.59 to 5.62); <0.001	1.63 (0.82 to 2.44); <0.001
PSM, 1:1 nearest-neighbor	108/108	4.23 (2.59 to 5.88); <0.001	2.89 (1.64 to 5.08); <0.001	4.28 (2.66 to 5.91); <0.001	1.80 (0.94 to 2.66); <0.001

For IPTW analyses, n refers to the original sample size before weighting. All sensitivity models were adjusted for age, sex, baseline PHQ-9 score, episode duration, first-episode status, comorbid anxiety, sleep problems, concurrent pharmacotherapy, family structure/family support indicator, follow-up duration, and the corresponding baseline WLVQ score.

After restricting the analysis to participants with follow-up duration within 6–10 weeks, the associations remained statistically significant for Δworldview (β = 5.00, 95% CI: 3.06 to 6.94; *p* < 0.001), worldview response (OR = 3.66, 95% CI: 1.87 to 7.18; *p* < 0.001), Δlife perspective (β = 3.91, 95% CI: 1.80 to 6.02; *p* < 0.001), and Δvalues total (β = 1.80, 95% CI: 0.83 to 2.77; *p* < 0.001). In IPTW and PSM analyses, the estimates were directionally consistent with those from the primary adjusted models. The maximum absolute SMD decreased from 0.247 before adjustment to 0.064 after IPTW and 0.085 after PSM, showing improved balance of measured covariates after propensity score adjustment (**Supplementary Table 5**).

In the continuous time-to-intervention analysis, each 7-day delay was associated with smaller changes in worldview (β = −1.32, 95% CI: −1.91 to −0.73; *p* < 0.001), life perspective (β = −1.14, 95% CI: −1.68 to −0.60; *p* < 0.001), and values total score (β = −0.58, 95% CI: −0.88 to −0.28; *p* < 0.001). Each 7-day delay was also associated with lower odds of response for worldview (OR = 0.76, 95% CI: 0.65 to 0.89; *p* = 0.001), life perspective (OR = 0.79, 95% CI: 0.68 to 0.92; *p* = 0.003), and values reconstruction (OR = 0.73, 95% CI: 0.59 to 0.91; *p* = 0.005) (**Supplementary Table 6**). These analyses describe observed associations in this retrospective cohort and should not be interpreted as evidence of causal effects.

## 4 Discussion

This retrospective cohort study provides real-world evidence that the timing of structured psychological intervention was associated with WLVQ-22-assessed reconstruction indicators in adolescents with depression. The novelty of this study lies in its focus on time-to-intervention as a clinically actionable service-delivery exposure, rather than only evaluating treatment type or symptom reduction. By examining worldview, life perspective, and values reconstruction, this study extends the outcome framework of adolescent depression beyond conventional affective symptom measures to exploratory cognitive–motivational and psychosocial domains. Earlier intervention initiation within a pragmatic 14-day clinical window was associated with greater improvements in worldview, life perspective, and values-related scores, and these associations were generally consistent across adjusted regression models, alternative timing thresholds, propensity score-based analyses, and continuous time-to-intervention analyses (23). These findings suggest that reducing delays in psychological intervention may be relevant to adolescent care pathways, particularly during the early phase of diagnostic clarification, family communication, treatment planning, and psychotherapy scheduling (24, 25). However, given the retrospective observational design and the exploratory nature of WLVQ-22-based outcomes, these findings should be interpreted as hypothesis-generating associations rather than evidence of causal benefit.

The findings for worldview and life perspective reconstruction provide insight into the cognitive–motivational dimensions that may be associated with earlier psychological engagement. Although baseline scores were comparable between groups, adolescents in the early-intervention group had higher follow-up scores, larger absolute and percent changes, and higher responder proportions for both domains. After adjustment for baseline symptom severity, episode duration, comorbid anxiety, sleep problems, pharmacotherapy, follow-up duration, and corresponding baseline WLVQ-22 scores, early intervention initiation remained associated with greater changes in worldview and life perspective. This pattern suggests that earlier entry into structured psychological care may be linked to more favorable short-term changes in meaning-making, perceived agency, future orientation, and goal-directed thinking. These constructs are clinically relevant because adolescent depression often involves pessimistic expectations, reduced controllability beliefs, impaired planning, and difficulties integrating negative experiences into a coherent self-narrative. The responder analyses further indicate that the between-group differences were not limited to mean-score changes, but were also reflected in a higher proportion of adolescents reaching prespecified improvement thresholds. Therefore, the worldview and life perspective findings support the value of assessing recovery-related cognitive and motivational domains alongside depressive symptom severity in adolescent clinical care (26, 27).

The values reconstruction findings further suggest that values-related recovery may be multidimensional rather than uniform across domains. The early-intervention group showed a higher follow-up total values score, a larger increase in the composite values score, and a higher values responder proportion. However, domain-specific analyses showed that the between-group differences were mainly driven by social connectedness and self-efficacy, whereas responsibility did not differ significantly between groups. This pattern may indicate that interpersonal reconnection and perceived personal competence are more sensitive to early therapeutic engagement during short-term follow-up, while responsibility-related behaviors may depend more heavily on sustained behavioral reinforcement, family expectations, school participation, and longer-term functional recovery. The borderline difference in achieving a follow-up values total score ≥60 also suggests that change-based outcomes may be more responsive than absolute attainment thresholds over a relatively short observation period. In addition, the continuous time-to-intervention analysis showed that each 7-day delay was associated with smaller changes in worldview, life perspective, and values total scores, as well as lower odds of response. This gradient-like pattern supports the relevance of considering intervention timing as a continuous service-process indicator, rather than relying solely on a single early-versus-delayed cutoff.

The present findings are broadly consistent with contemporary guidance and evidence syntheses emphasizing timely access to evidence-based psychological care for youth depression. The 2023 AACAP Clinical Practice Guideline identifies psychotherapy, particularly cognitive-behavioral and interpersonal therapies, and SSRIs as evidence-supported treatment options, while also emphasizing comprehensive assessment and individualized care planning (28, 29). Although current guidelines do not define intervention timing as a primary therapeutic parameter, our study addresses a clinically relevant service-delivery question: whether earlier initiation of structured psychological intervention is associated with more favorable short-term functional–cognitive reconstruction outcomes under routine clinical conditions. This interpretation is also aligned with a recent systematic review showing that early intervention models for common youth mental health problems may improve access, experience, and outcomes, while emphasizing heterogeneity and the need for more robust controlled studies (30). By focusing on time-to-intervention as the exposure, the present study extends prior work beyond treatment type and symptom reduction to WLVQ-22-assessed exploratory indicators of worldview, future orientation, and values-related reconstruction. This is clinically relevant given longitudinal evidence linking adolescent depressive symptoms to poorer psychosocial outcomes in young adulthood, including reduced social support (31). Moreover, recent evidence updates and meta-analyses indicate that psychosocial interventions for adolescents can produce measurable, although often modest, improvements in depressive symptoms, particularly when delivered as targeted interventions by trained professionals (32, 33). Together, these findings support the relevance of timely entry into structured psychological care pathways while underscoring the need for prospective studies to evaluate intervention timing more directly (34).

A cautious interpretation of these findings is that earlier psychological intervention may provide structured cognitive and interpersonal support during a developmental period when self-concept, future orientation, and value-related beliefs remain relatively malleable. From cognitive-behavioral and developmental perspectives, earlier engagement may be associated with earlier opportunities for cognitive restructuring, behavioral activation, problem-solving, interpersonal support, and values clarification, which may partly explain the more favorable WLVQ-22-assessed reconstruction indicators observed in the early-intervention group. The findings may also have implications for adolescents with co-occurring clinical concerns, as comorbid anxiety, sleep problems, and concurrent pharmacotherapy were considered in the adjusted analyses, and adolescents with depression often present with overlapping anxiety symptoms, sleep disturbance, interpersonal withdrawal, reduced agency, and functional difficulties. Improvements in worldview, life perspective, and values reconstruction may further reflect broader well-being-related domains, including perceived meaning, future orientation, social connectedness, responsibility, and self-efficacy (35). However, because this study was retrospective and observational, these interpretations should be regarded as hypothesis-generating rather than mechanistic evidence, and broader psychological well-being was not directly measured using validated well-being or functional outcome scales.

Several limitations should be acknowledged. First, because this was a retrospective observational study, the observed associations cannot establish causality. Although adjusted regression models, IPTW, PSM, and sensitivity analyses were used to reduce measured confounding and showed generally consistent results, residual or unmeasured confounding may still have influenced both intervention timing and reconstruction outcomes. Second, exposure misclassification is possible if psychological intervention initiation dates were recorded inaccurately, and differences in treatment implementation may not have been fully captured by the timing variable alone. Third, the 14-day cutoff was selected as a pragmatic early-care definition rather than a validated critical window for cognitive–motivational reconstruction. The attenuated responder association under the stricter 7-day cutoff suggests possible threshold dependence, and future prospective studies should evaluate intervention timing both as a continuous exposure and across clinically meaningful time windows. Fourth, the WLVQ-22 has not been externally validated in independent adolescent depression cohorts. Although exploratory analyses in this cohort showed preliminary internal consistency, convergent validity, and responsiveness, WLVQ-22-based outcomes should be interpreted as exploratory reconstruction indicators rather than fully validated clinical endpoints. Fifth, the follow-up period captured only short-term reconstruction and may not reflect the durability of change. Longer-term studies are needed to determine whether timing-related differences persist over time. Sixth, generalizability is limited by the single-center design and the Chinese sociocultural and healthcare-system context, as worldview, life perspective, values reconstruction, psychotherapy access, and early-care pathways may vary across settings. Finally, multiple outcomes may increase the probability of chance findings, although the consistency across domains, adjusted models, and sensitivity analyses supports the robustness of the observed pattern. Future multicenter, cross-cultural, prospective studies using standardized follow-up schedules, blinded assessment where feasible, and externally validated instruments are warranted.

## 5 Conclusion

In adolescents with depression, both early- and delayed-intervention groups showed significant improvements in worldview, life perspective, and values reconstruction at follow-up. Earlier initiation was consistently associated with greater absolute and percent gains and higher responder rates. These associations persisted after multivariable adjustment and were generally robust across sensitivity analyses, suggesting the potential clinical relevance of reducing delays to structured psychological care while warranting confirmation in prospective studies.

## Data Availability

The raw data supporting the conclusions of this article will be made available by the authors, without undue reservation.

## References

[1] Rikard-Bell C Hunt C McAulay C Hay P Morad A Cunich M . Adolescent depression from a developmental perspective: The importance of recognizing developmental distress in depressed adolescents. Int J Environ Res Public Health. (2022) 19:16029. doi: 10.3390/ijerph192316029 36498102 PMC9736143

[2] Xiang AH Martinez MP Chow T Carter SA Negriff S Velasquez B . Depression and anxiety among US children and young adults. JAMA Netw Open. (2024) 7:e2436906. doi: 10.1001/jamanetworkopen.2024.36906 39352699 PMC11445688

[3] Wu P Kong L Zhou Y Deng C Wang Z Shen Y . Neurobiological subtypes of adolescent depression: A multimodal integration of morphometric similarity network and spatial transcriptomics. Mol Psychiatry. (2025) 30:5448–62. doi: 10.1038/s41380-025-03133-7 40753308 PMC12532574

[4] Liu Z Kuai M . The global burden of depression in adolescents and young adults, 1990-2021: Systematic analysis of the global burden of disease study. BMC Psychiatry. (2025) 25:767. doi: 10.1186/s12888-025-07201-8 40770320 PMC12326784

[5] Tian J Yan N Hu X Tian S Wang Y Mackay LE . Global burden of mental disorders among adolescents and young adults, 1990-2021: A systematic analysis of the Global Burden of Diseases Study 2021. Gen Psychiatr. (2025) 38:e102278. doi: 10.1136/gpsych-2025-102278 41451038 PMC12730781

[6] Lu B Lin L Su X . Global burden of depression or depressive symptoms in children and adolescents: A systematic review and meta-analysis. J Affect Disord. (2024) 354:553–62. doi: 10.1016/j.jad.2024.03.074 38490591

[7] Rao WW Zhang JW Zong QQ An FR Ungvari GS Balbuena L . Prevalence of depressive symptoms in overweight and obese children and adolescents in mainland China: A meta-analysis of comparative studies and epidemiological surveys. J Affect Disord. (2019) 250:26–34. doi: 10.1016/j.jad.2019.02.045 30826491

[8] Defayette AB Whitmyre ED López R Jr. Brown B Wolff JC Spirito A . Adolescent depressed mood and difficulties with emotion regulation: Concurrent trajectories of change. J Adolesc. (2021) 91:1–14. doi: 10.1016/j.adolescence.2021.07.001 34252783 PMC8380718

[9] Zhang BR Tian SY . Social cognition in adolescent depression: Evolution of research content and future perspectives. World J Psychiatry. (2025) 15:110598. doi: 10.5498/wjp.v15.i10.110598 41112609 PMC12531973

[10] Tang P Kostyrka-Allchorne K Butura AM Phillips-Owen J Sonuga-Barke E . Reciprocal developmental pathways between future-related thinking and symptoms of adolescent depression and anxiety: A systematic review and meta-analysis of longitudinal studies. Clin Psychol Rev. (2024) 112:102465. doi: 10.1016/j.cpr.2024.102465 39002184

[11] Sharrad KJ Sanwo O Cuevas-Asturias S Kew KM Carson-Chahhoud KV Pike KC . Psychological interventions for asthma in children and adolescents. Cochrane Database Syst Rev. (2024) 1:Cd013420. doi: 10.1002/14651858.CD013420.pub2 38205864 PMC10782779

[12] Li Y Kwok S . Characteristics and correlates of meaning in life profiles among Chinese rural adolescents. J Psychol. (2024) 158:627–49. doi: 10.1080/00223980.2024.2338760 38652653

[13] Kwok S Fang S Huang BM Addis Tesfaw A Deng X . Stability and changes in meaning in life profiles and their impact on mental health among chinese university students: A latent transition analysis. Front Psychol. (2025) 16:1529851. doi: 10.3389/fpsyg.2025.1529851 39981384 PMC11840567

[14] Korczak DJ Westwell-Roper C Sassi R . Diagnosis and management of depression in adolescents. Cmaj. (2023) 195:E739–e46. doi: 10.1503/cmaj.220966 37247881 PMC10228578

[15] Yu X Zhao B Yin T Qu H Zhang J Cheng X . Effect of acceptance and commitment therapy for adolescent depression: A meta-analysis. Front Psychiatry. (2025) 16:1506822. doi: 10.3389/fpsyt.2025.1506822 40458781 PMC12128421

[16] Chen B Li J Qi Y Mao H Liu Y Wang W . Comparative effectiveness and acceptability of internet-based psychological interventions on depression in young people: A systematic review and network meta-analysis. BMC Psychiatry. (2025) 25:321. doi: 10.1186/s12888-025-06757-9 40175933 PMC11967053

[17] Peake E Miller I Flannery J Chen L Lake J Padmanabhan A . Preliminary efficacy of a digital intervention for adolescent depression: Randomized controlled trial. J Med Internet Res. (2024) 26:e48467. doi: 10.2196/48467 38324367 PMC10882470

[18] Zhao Y Li Y Zhao J . A qualitative study of the reasons for delayed medical treatment in adolescents with depression based on the health ecology model. Front Public Health. (2023) 11:1124397. doi: 10.3389/fpubh.2023.1124397 37559744 PMC10407104

[19] Peñacoba-Puente C Rodríguez-Rojo IC Luengo-González R González-Alegre P Cuesta-Lozano D Asenjo-Esteve Á . Positive mental health in adolescents: Implications of cognitive–emotional processes and social support. Soc Sci. (2025) 14:461. doi: 10.3390/socsci1408046130654563

[20] Brown FL Lee C Servili C Willhoite A Van Ommeren M Hijazi Z . Psychological interventions for children with emotional and behavioral difficulties aged 5-12 years: An evidence review. Glob Ment Health (Camb). (2024) 11:e75. doi: 10.1017/gmh.2024.57 39314997 PMC11418085

[21] Méndez J Sánchez-Hernández Ó Garber J Espada JP Orgilés M . Psychological treatments for depression in adolescents: More than three decades later. Int J Environ Res Public Health. (2021) 18:4600. doi: 10.3390/ijerph18094600 33926111 PMC8123571

[22] Cao P Li Y Yang L Guo L Hua J Zhang S . Evidence for various interventions to reduce depressive symptoms for children and adolescents: Protocol of a global evidence and gap map. Syst Rev. (2025) 14:162. doi: 10.1186/s13643-025-02909-w 40781690 PMC12333166

[23] Amoah J Stuart EA Cosgrove SE Harris AD Han JH Lautenbach E . Comparing propensity score methods versus traditional regression analysis for the evaluation of observational data: A case study evaluating the treatment of gram-negative bloodstream infections. Clin Infect Dis. (2020) 71:e497–505. doi: 10.1093/cid/ciaa169 32069360 PMC7713675

[24] Widyasih H Yunita S Adnani QES Arisanti N Pandia V Dhamayanti M . A scoping review of family-based prevention strategies in adolescent mental health. Psychol Res Behav Manag. (2025) 18:2429–50. doi: 10.2147/prbm.S568366 41480334 PMC12753846

[25] O'Dea B McNamara R Ma IC Gu A Jiang F Milesi J . Addressing the psychological needs of adolescents during the wait time for mental health treatment: Service design study. JMIR Form Res. (2026) 10:e87067. doi: 10.2196/87067 41951218 PMC13184595

[26] Uhlhaas PJ Davey CG Mehta UM Shah J Torous J Allen NB . Towards a youth mental health paradigm: A perspective and roadmap. Mol Psychiatry. (2023) 28:3171–81. doi: 10.1038/s41380-023-02202-z 37580524 PMC10618105

[27] Karcher NR Hicks R Schiffman J Asarnow JR Calkins ME Dauberman JL . Youth mental health screening and linkage to care. Psychiatr Serv. (2023) 74:727–36. doi: 10.1176/appi.ps.202200008 36695011 PMC10329990

[28] Walter HJ Abright AR Bukstein OG Diamond J Keable H Ripperger-Suhler J . Clinical practice guideline for the assessment and treatment of children and adolescents with major and persistent depressive disorders. J Am Acad Child Adolesc Psychiatry. (2023) 62:479–502. doi: 10.1016/j.jaac.2022.10.001 36273673

[29] Machmutow K Meister R Jansen A Kriston L Watzke B Härter MC . Comparative effectiveness of continuation and maintenance treatments for persistent depressive disorder in adults. Cochrane Database Syst Rev. (2019) 5:Cd012855. doi: 10.1002/14651858.CD012855.pub2 31106850 PMC6526465

[30] Appleton R Barnett P Clarke C Yang J Begum S Edbrooke-Childs J . Approaches to early intervention for common mental health problems in young people: A systematic review. BMC Med. (2025) 23:651. doi: 10.1186/s12916-025-04438-8 41286829 PMC12642248

[31] Psychogiou L Navarro MC Orri M Côté SM Ahun MN . Childhood and adolescent depression symptoms and young adult mental health and psychosocial outcomes. JAMA Netw Open. (2024) 7:e2425987. doi: 10.1001/jamanetworkopen.2024.25987 39115846 PMC11310820

[32] Weersing VR Goger P Schwartz KTG Baca SA Angulo F Kado-Walton M . Evidence-base update of psychosocial and combination treatments for child and adolescent depression. J Clin Child Adolesc Psychol. (2025) 54:1–51. doi: 10.1080/15374416.2024.2384022 39495037

[33] Weersing VR Jeffreys M Do MT Schwartz KT Bolano C . Evidence base update of psychosocial treatments for child and adolescent depression. J Clin Child Adolesc Psychol. (2017) 46:11–43. doi: 10.1080/15374416.2016.1220310 27870579 PMC5296370

[34] Duagi D Carter B Farrelly M Lisk S Shearer J Byford S . Long-term effects of psychosocial interventions for adolescents on depression and anxiety: A systematic review and meta-analysis. EClinicalMedicine. (2024) 68:102382. doi: 10.1016/j.eclinm.2023.102382 38273890 PMC10809118

[35] Solberg H Idsoe T Keles S . Reducing anxiety symptoms in adolescents with pre-existing depression: Results from a randomized control trial. Front Psychiatry. (2025) 16. doi: 10.3389/fpsyt.2025.1461887 40612034 PMC12222114

