# Supplementary material for: Association between timing of psychological intervention and reconstruction of worldview, life perspective, and values in adolescents with depression
Source: Front Psychiatry. 2026 Oct 5;17:1786690. doi: 10.3389/fpsyt.2026.1786690 (PMC13678166; doi:10.3389/fpsyt.2026.1786690)
Supplement: Supplementary file 1 [file Table1.docx]

Supplementary Table 1. Worldview Reconstruction Questionnaire (WLVQ-WV, 5 items)

| Subscale | Item code | Item statement (past 2 weeks) | Response options (0–4) | Reverse-coded |
| --- | --- | --- | --- | --- |
| Meaning/coherence | WV1 | I could make sense of what was happening in my life. | 0 not at all; 1 rarely; 2 sometimes; 3 often; 4 almost always | No |
| Meaning/coherence | WV2 | I could find at least some meaning in daily experiences. | 0 not at all; 1 rarely; 2 sometimes; 3 often; 4 almost always | No |
| Safety/trust | WV3 | I felt that the world around me was mostly unsafe. | 0 not at all; 1 rarely; 2 sometimes; 3 often; 4 almost always | Yes |
| Agency/controllability | WV4 | I felt that nothing I did could change my situation. | 0 not at all; 1 rarely; 2 sometimes; 3 often; 4 almost always | Yes |
| Balance/acceptance | WV5 | I could hold positive and negative experiences together without feeling overwhelmed. | 0 not at all; 1 rarely; 2 sometimes; 3 often; 4 almost always | No |

Notes: The Worldview total score is computed as the mean of the 5 items after reverse-coding, transformed to a 0–100 scale as (mean item score/4) × 100. Absolute change is defined as Δ = follow-up − baseline. Responder status is defined as Δ ≥ 10 points.

Abbreviations: Δ, absolute change from baseline to follow-up; WLVQ-WV, Worldview Reconstruction subscale of the Worldview, Life Perspective, and Values Reconstruction Questionnaire.

Supplementary Table 2. Life Perspective Reconstruction Questionnaire (WLVQ-LP, 5 items)

| Subscale | Item code | Item statement (past 2 weeks) | Response options (0–4) | Reverse-coded |
| --- | --- | --- | --- | --- |
| Future orientation | LP1 | I could imagine a realistic future for myself. | 0 not at all; 1 rarely; 2 sometimes; 3 often; 4 almost always | No |
| Goal clarity | LP2 | I had clear goals for school or personal life. | 0 not at all; 1 rarely; 2 sometimes; 3 often; 4 almost always | No |
| Planning | LP3 | I had a workable plan for the next few weeks or months. | 0 not at all; 1 rarely; 2 sometimes; 3 often; 4 almost always | No |
| Hopelessness | LP4 | I felt that my future would not improve no matter what I did. | 0 not at all; 1 rarely; 2 sometimes; 3 often; 4 almost always | Yes |
| Problem-solving orientation | LP5 | When difficulties arose, I could think of at least one step I could take. | 0 not at all; 1 rarely; 2 sometimes; 3 often; 4 almost always | No |

Notes: The Life Perspective total score is computed as the mean of the 5 items after reverse-coding, transformed to a 0–100 scale as (mean item score/4) × 100. Absolute change is defined as Δ = follow-up − baseline. Responder status is defined as Δ ≥ 10 points.

Abbreviations: Δ, absolute change from baseline to follow-up; WLVQ-LP, Life Perspective Reconstruction subscale of the Worldview, Life Perspective, and Values Reconstruction Questionnaire.

Supplementary Table 3. Values Reconstruction Questionnaire (WLVQ-V, 12 items)

| Domain | Item code | Item statement (past 2 weeks) | Response options (0–4) | Reverse-coded |
| --- | --- | --- | --- | --- |
| Responsibility | VR1 | I took responsibility for my actions, including mistakes. | 0 not at all; 1 rarely; 2 sometimes; 3 often; 4 almost always | No |
| Responsibility | VR2 | I completed expected tasks (e.g., homework/chores) on time. | 0 not at all; 1 rarely; 2 sometimes; 3 often; 4 almost always | No |
| Responsibility | VR3 | I avoided responsibilities even when they were important. | 0 not at all; 1 rarely; 2 sometimes; 3 often; 4 almost always | Yes |
| Responsibility | VR4 | I tried to keep commitments I made to others. | 0 not at all; 1 rarely; 2 sometimes; 3 often; 4 almost always | No |
| Social connectedness | VS1 | I felt connected to at least one person who understood me. | 0 not at all; 1 rarely; 2 sometimes; 3 often; 4 almost always | No |
| Social connectedness | VS2 | I withdrew from others even when support was available. | 0 not at all; 1 rarely; 2 sometimes; 3 often; 4 almost always | Yes |
| Social connectedness | VS3 | I communicated my feelings to someone I trusted. | 0 not at all; 1 rarely; 2 sometimes; 3 often; 4 almost always | No |
| Social connectedness | VS4 | I participated in family, school, or peer activities at least occasionally. | 0 not at all; 1 rarely; 2 sometimes; 3 often; 4 almost always | No |
| Self-efficacy | VE1 | I believed I could handle a difficult task if I tried. | 0 not at all; 1 rarely; 2 sometimes; 3 often; 4 almost always | No |
| Self-efficacy | VE2 | When I had a problem, I could find a way to solve it. | 0 not at all; 1 rarely; 2 sometimes; 3 often; 4 almost always | No |
| Self-efficacy | VE3 | I felt incapable of coping with daily demands. | 0 not at all; 1 rarely; 2 sometimes; 3 often; 4 almost always | Yes |
| Self-efficacy | VE4 | I used at least one strategy to manage distress (e.g., breathing, journaling, seeking help). | 0 not at all; 1 rarely; 2 sometimes; 3 often; 4 almost always | No |

Notes: Each domain score (responsibility, social connectedness, self-efficacy) is computed as the mean of its 4 items after reverse-coding, transformed to a 0–100 scale as (mean/4) × 100. The Values total score is the arithmetic mean of the three domain scores, which matches the Results reporting. Absolute change is defined as Δ = follow-up − baseline. Responder status is defined as Δvalues total ≥ 10 points. Domain-level changes (Δresponsibility, Δsocial connectedness, Δself-efficacy) are computed analogously to support ranking of improvements.

Abbreviations: Δ, absolute change from baseline to follow-up; WLVQ-V, Values Reconstruction section of the Worldview, Life Perspective, and Values Reconstruction Questionnaire.

Supplementary Table S4. Psychometric properties of the WLVQ-22

| Scale/domain | No. of items | Cronbach’s α | Corrected item-total correlation, range | Correlation with PHQ-9 at baseline, r | *p* value | Standardized response mean |
| --- | --- | --- | --- | --- | --- | --- |
| Worldview reconstruction | 5 | 0.84 | 0.46 to 0.71 | −0.52 | <0.001 | 1.71 |
| Life perspective reconstruction | 5 | 0.86 | 0.49 to 0.74 | −0.55 | <0.001 | 1.55 |
| Values reconstruction, total | 12 | 0.91 | 0.43 to 0.76 | −0.48 | <0.001 | 2.15 |
| Responsibility | 4 | 0.78 | 0.41 to 0.63 | −0.36 | <0.001 | 1.34 |
| Social connectedness | 4 | 0.82 | 0.47 to 0.69 | −0.44 | <0.001 | 1.39 |
| Self-efficacy | 4 | 0.80 | 0.45 to 0.66 | −0.46 | <0.001 | 1.28 |
| WLVQ-22 total score | 22 | 0.93 | 0.40 to 0.78 | −0.57 | <0.001 | 1.88 |

Supplementary Table S5. Covariate balance before and after IPTW and PSM

| Covariate | Before adjustment Absolute SMD | After IPTW Absolute SMD | After PSM Absolute SMD |
| --- | --- | --- | --- |
| Age, years | 0.247 | 0.041 | 0.052 |
| Male sex | 0.147 | 0.026 | 0.044 |
| Senior grade | 0.212 | 0.058 | 0.071 |
| Family structure | 0.240* | 0.064* | 0.085* |
| PHQ-9 score | 0.064 | 0.018 | 0.033 |
| Episode duration, months | 0.086† | 0.022 | 0.028 |
| First episode | 0.214 | 0.052 | 0.069 |
| Comorbid anxiety | 0.071 | 0.031 | 0.038 |
| Sleep problems | 0.141 | 0.036 | 0.047 |
| Concomitant pharmacotherapy | 0.024 | 0.013 | 0.021 |
| Medication class among treated | 0.141* | 0.043* | 0.055* |
| Fluoxetine-equivalent dose, mg/day‡ | 0.192 | 0.049 | 0.061 |
| Follow-up duration, weeks | 0.026 | 0.015 | 0.022 |
| Baseline worldview score | 0.146 | 0.025 | 0.031 |
| Baseline life perspective score | 0.140 | 0.028 | 0.035 |
| Baseline values total score | 0.062 | 0.017 | 0.024 |
| Maximum absolute SMD | 0.247 | 0.064 | 0.085 |

SMD, standardized mean difference; IPTW, inverse probability of treatment weighting; PSM, propensity score matching. An absolute SMD <0.10 was considered indicative of acceptable balance.

*For multi-category variables, the reported value represents the largest category-specific absolute SMD.

†For episode duration, the before-adjustment SMD was calculated using individual-level data in the propensity score balance diagnostics; therefore, it differs from Table 1, where SMD was not estimated from summary median and IQR values.

‡Medication class and fluoxetine-equivalent dose were evaluated among participants receiving concomitant pharmacotherapy and are presented as treatment-subgroup balance indicators.

Supplementary Table S6. Continuous time-to-intervention analysis per 7-day delay

| Outcome | Model type | Effect estimate per 7-day delay | 95% CI | *p* value |
| --- | --- | --- | --- | --- |
| Δworldview | Linear regression | β = −1.32 | −1.91 to −0.73 | <0.001 |
| Δlife perspective | Linear regression | β = −1.14 | −1.68 to −0.60 | <0.001 |
| Δvalues total | Linear regression | β = −0.58 | −0.88 to −0.28 | <0.001 |
| Worldview responder | Logistic regression | OR = 0.76 | 0.65 to 0.89 | 0.001 |
| Life perspective responder | Logistic regression | OR = 0.79 | 0.68 to 0.92 | 0.003 |
| Values responder | Logistic regression | OR = 0.73 | 0.59 to 0.91 | 0.005 |
